# The value management cycle as a sustainable HRM framework: insights from Chinese Huawei’s practices of value creation, evaluation, and distribution

**DOI:** 10.3389/fpsyg.2026.1825744

**Published:** 2026-05-18

**Authors:** Rongcheng Liang

**Affiliations:** 1 Emergency Management Training Department, Party School of Shandong Provincial Committee of the Communist Party of China (Shandong Academy of Governance), Jinan, China; 2 School of International Relations and Public Affairs, Fudan University, Shanghai, China

**Keywords:** sustainable HRM, value creation, value distribution, value evaluation, value management cycle

## Abstract

This study develops a theoretical framework for sustainable human resource management (HRM) based on the value management cycle, comprising value creation, evaluation, and distribution. Drawing upon extensive case analysis of Huawei’s pioneering HRM practices, this study adopts a qualitative single-case study methodology drawing on multiple data sources, including document analysis (1996–2024), semi-structured interviews (2020–2023), direct observation, and secondary data, and employs pattern matching and explanation building techniques for data analysis. This research examines how a closed-loop value management system can foster organizational sustainability by aligning employee interests with long-term strategic objectives. The proposed framework integrates three core principles: customer-centric value creation, result-oriented value evaluation, and striver-focused value distribution. Our analysis reveals that Huawei’s value management cycle, established in the late 1990s and continuously refined over two decades, has enabled the company to effectively manage nearly 200,000 knowledge workers while maintaining high levels of organizational vitality and innovation capacity. The findings demonstrate that sustainable HRM requires not only individual HR practice optimization but also systematic integration of value creation, objective evaluation, and equitable distribution mechanisms. This study contributes to the sustainable HRM literature by providing an actionable framework derived from one of China’s most successful technology enterprises, offering practical insights for organizations seeking to balance employee well-being with competitive performance in the knowledge economy era.

## 1 Introduction

The concept of sustainable human resource management (HRM) has gained significant scholarly attention in recent years as organizations grapple with the dual challenges of maintaining competitive advantage while ensuring employee well-being and long-term organizational viability (Amin and Rahman, 2026; Siddique et al., 2026). Unlike traditional HRM approaches that primarily focus on short-term performance optimization, sustainable HRM emphasizes the development of human capital in ways that support both current operational needs and future organizational resilience (Aust et al., 2020). This paradigm shift reflects a growing recognition that human resources represent not merely costs to be controlled but strategic assets requiring sustained investment and development.

The theoretical foundations of sustainable HRM draw from multiple disciplinary perspectives, including the Resource-Based View (RBV) of the firm, stakeholder theory, and institutional theory (Stankevičiūtė and Savanevičienė, 2018). From an RBV perspective, human capital constitutes a source of sustainable competitive advantage when it is valuable, rare, inimitable, and non-substitutable (Barney, 1991). Sustainable HRM practices aim to cultivate such human capital by fostering employee competencies, motivation, and commitment in ways that transcend short-term transactional exchanges (Ullah et al., 2026). However, despite the growing body of literature on sustainable HRM, significant gaps remain in understanding how organizations can effectively implement integrated HRM systems that balance multiple stakeholder interests while maintaining operational efficiency (Faisal and Naushad, 2020). Existing research has often examined individual HRM practices in isolation, such as green HRM, ethical leadership, or employee well-being initiatives, without adequately addressing how these practices can be systematically integrated into a coherent framework (Katper et al., 2026; Anlesinya and Susomrith, 2020).

This study addresses this research gap by introducing the value management cycle as a comprehensive framework for sustainable HRM. The value management cycle, comprising three interconnected components—value creation, value evaluation, and value distribution—provides a theoretically grounded and practically proven approach to aligning organizational and employee interests in pursuit of sustainable competitive advantage. This framework, originally developed and implemented by Huawei Technologies in the late 1990s, has enabled the company to grow from a small telecommunications equipment trader to one of the world’s leading technology enterprises with nearly 200,000 employees across more than 170 countries. Huawei’s value management system represents a particularly valuable case for academic study because it demonstrates how a Chinese enterprise successfully adapted Western management theories to create an innovative HRM model that addresses the unique challenges of managing knowledge workers in a high-technology industry. The company’s approach, crystallized in the famous “Huawei Basic Law” drafted by Professor Jianfeng Peng and his consulting team in 1998, established the principle that “knowledge workers are the primary creators of value” and designed the entire HRM system around this fundamental premise.

The research questions guiding this study are: (1) How does the value management cycle function as an integrated system for sustainable HRM? (2) What specific mechanisms enable value creation, evaluation, and distribution to reinforce each other in generating sustainable organizational outcomes? (3) What lessons can other organizations derive from Huawei’s implementation experience? This study makes several contributions to the sustainable HRM literature. First, it provides a theoretically grounded framework that integrates fragmented sustainable HRM research into a coherent system. Second, it offers detailed empirical insights from one of the world’s most successful technology companies, demonstrating how sustainable HRM principles can be operationalized at scale. Third, it develops practical recommendations for organizations seeking to implement value-based HRM systems.

## 2 Theoretical framework

### 2.1 Sustainable human resource management

Sustainable HRM emerged as a distinct research field in the early 2000s, reflecting growing concerns about the long-term consequences of short-term oriented HRM practices that prioritize immediate performance over employee well-being and development (Amin and Rahman, 2026). Drawing inspiration from the broader concept of sustainable development, sustainable HRM seeks to balance economic, social, and environmental objectives in the management of human resources (Siddique et al., 2026).

Aust et al. (2020) identified four distinct categories of sustainable HRM: (1) socially responsible HRM, which focuses on generating effective results across social and economic domains; (2) green HRM, which emphasizes environmental sustainability alongside economic performance; (3) triple bottom line HRM, which integrates economic, social, and environmental considerations; and (4) common good HRM, which harnesses human resources to promote values benefiting society at large. These categories share a common emphasis on viewing HRM not merely as a tool for achieving short-term business objectives but as a strategic function contributing to long-term organizational and societal sustainability.

The mechanisms through which sustainable HRM influences organizational outcomes have been conceptualized through various theoretical lenses. Social Exchange Theory (SET) suggests that when organizations invest in employee well-being and development, employees reciprocate with increased commitment, effort, and citizenship behaviors (Cropanzano and Mitchell, 2005). The Ability-Motivation-Opportunity (AMO) framework posits that sustainable HRM enhances employee performance by developing abilities, fostering motivation, and providing opportunities for contribution (Rifqi, 2026). Recent empirical research has provided evidence supporting the positive effects of sustainable HRM practices on various outcomes. Katper et al. (2026) found that sustainable HRM bundles positively influence employee well-being and organizational performance. Stankevičiūtė and Savanevičienė (2018) demonstrated that sustainable HRM reduces work-related stress and burnout while enhancing employee engagement. These findings suggest that sustainable HRM represents not merely an ethical imperative but a strategic approach to achieving competitive advantage through human capital development.

### 2.2 The value management cycle framework

**Figure 1:**
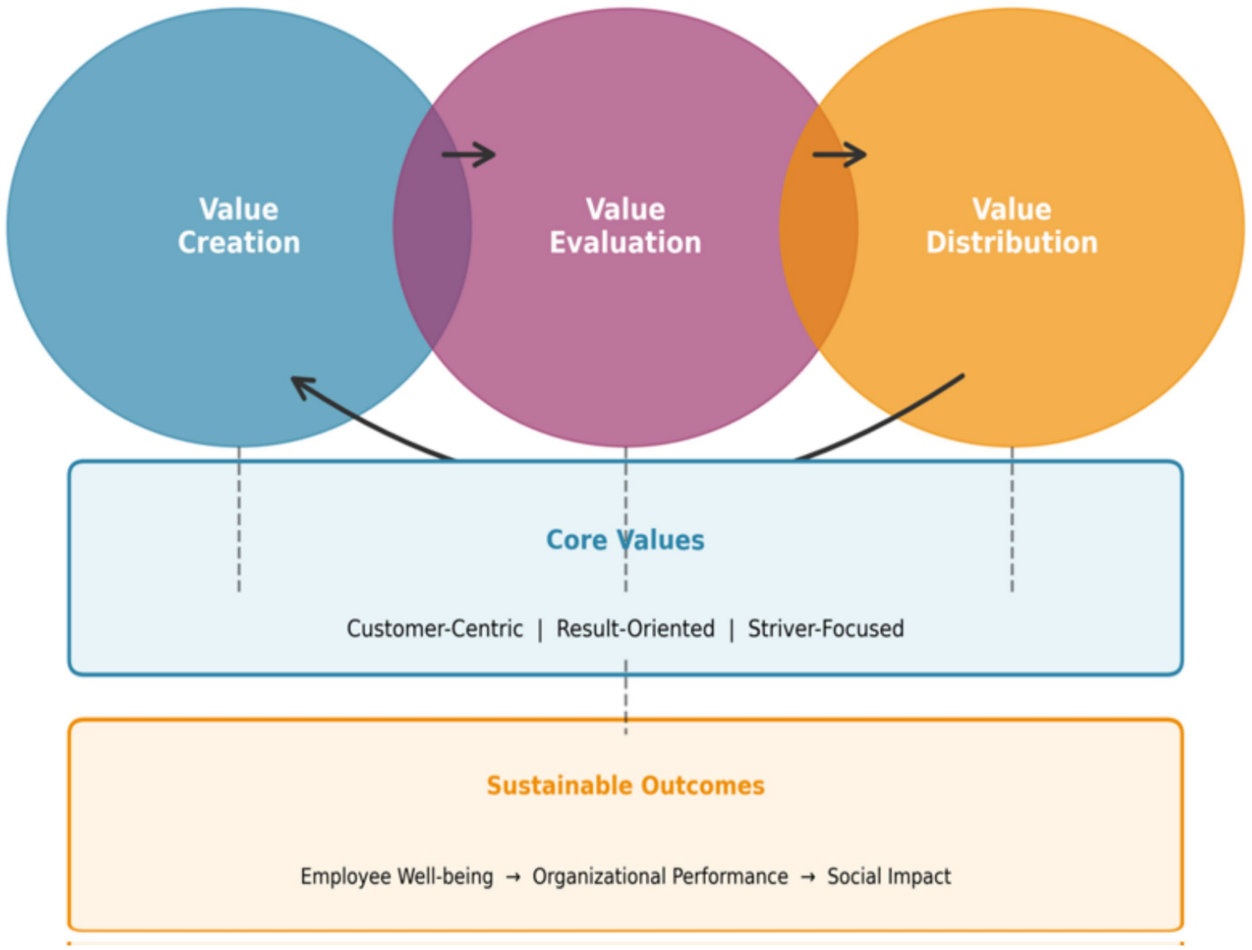
The value management cycle framework.

The value management cycle represents an integrated approach to HRM that views human resource activities as interconnected components of a closed-loop system rather than isolated functional practices. This framework, grounded in systems theory and value chain analysis, conceptualizes HRM as a continuous cycle comprising three core processes: value creation, value evaluation, and value distribution (see Figure 1).

Value creation addresses the fundamental question of how organizations generate value through human resource deployment. In the context of HRM, value creation involves designing work systems, organizational structures, and cultural environments that enable employees to contribute maximally to organizational objectives (Porter, 1985). The value creation perspective shifts HRM focus from cost minimization to value maximization, emphasizing the development of human capital as a primary source of competitive advantage.

Value evaluation provides the measurement and assessment mechanisms necessary for determining the relative contributions of different organizational elements to overall value creation. Effective evaluation systems must be objective, comprehensive, and aligned with strategic objectives to ensure that value assessments accurately reflect true contributions. In HRM contexts, value evaluation encompasses position evaluation, performance assessment, and competency appraisal, each addressing different dimensions of employee contribution.

Value distribution addresses how the value created by the organization is allocated among various stakeholders, particularly employees. Equitable distribution mechanisms ensure that contributors receive proportionate rewards, thereby maintaining motivation and reinforcing desired behaviors. The distribution system serves as a powerful signaling mechanism, communicating organizational priorities and expectations to employees while providing tangible recognition for contributions.

The three components of the value management cycle form an integrated system in which each element influences and reinforces the others. Value creation provides the foundation for evaluation, which in turn enables fair distribution. Distribution outcomes subsequently influence future value creation by shaping employee motivation and behavior. This cyclical relationship creates a self-reinforcing system that, when properly designed and implemented, can generate sustainable competitive advantage through continuous human capital development and motivation.

## 3 Research methodology

This study employs a qualitative case study methodology to examine how the value management cycle functions as a sustainable HRM framework in practice. Case study research is particularly appropriate for investigating complex organizational phenomena within their real-life contexts, especially when the boundaries between phenomenon and context are not clearly evident (Yin, 2018). The single-case design is justified by the revelatory nature of Huawei’s value management system, which represents a critical case for testing and developing sustainable HRM theory.

Data collection for this study involved multiple sources to ensure triangulation and enhance validity. Primary data sources include: (1) documentary analysis of Huawei’s official publications, including the Huawei Basic Law, annual reports, and management documents spanning the period from 1996 to 2024; (2) in-depth interviews with current and former Huawei executives and HR professionals conducted between 2020 and 2023; (3) direct observation of HR practices during consulting engagements; and (4) analysis of secondary sources including academic publications, business case studies, and media reports.

Data analysis followed an iterative process of pattern matching and explanation building. Initial coding identified key themes related to value creation, evaluation, and distribution practices. Subsequent analysis examined the relationships between these themes and their connections to sustainable HRM outcomes. The analysis was guided by the theoretical framework developed in Section 2, while remaining open to emergent findings that might extend or refine existing theory.

To enhance the rigor of the research, several strategies were employed. First, multiple data sources were used to corroborate findings and reduce reliance on any single source. Second, key informants reviewed preliminary findings to ensure accuracy and completeness. Third, the research team maintained detailed documentation of analytical decisions to enable audit trails. Fourth, the analysis explicitly considered alternative explanations for observed patterns.

## 4 Findings: Huawei’s value management cycle in practice

Huawei’s value management system, developed over nearly three decades of organizational evolution, provides a comprehensive illustration of how the value management cycle can function as a sustainable HRM framework. The following sections examine each component of the cycle in detail, drawing on documentary evidence and interview data to illustrate key mechanisms and their interconnections.

### 4.1 Value creation: customer-centric value generation

**Figure 2:**
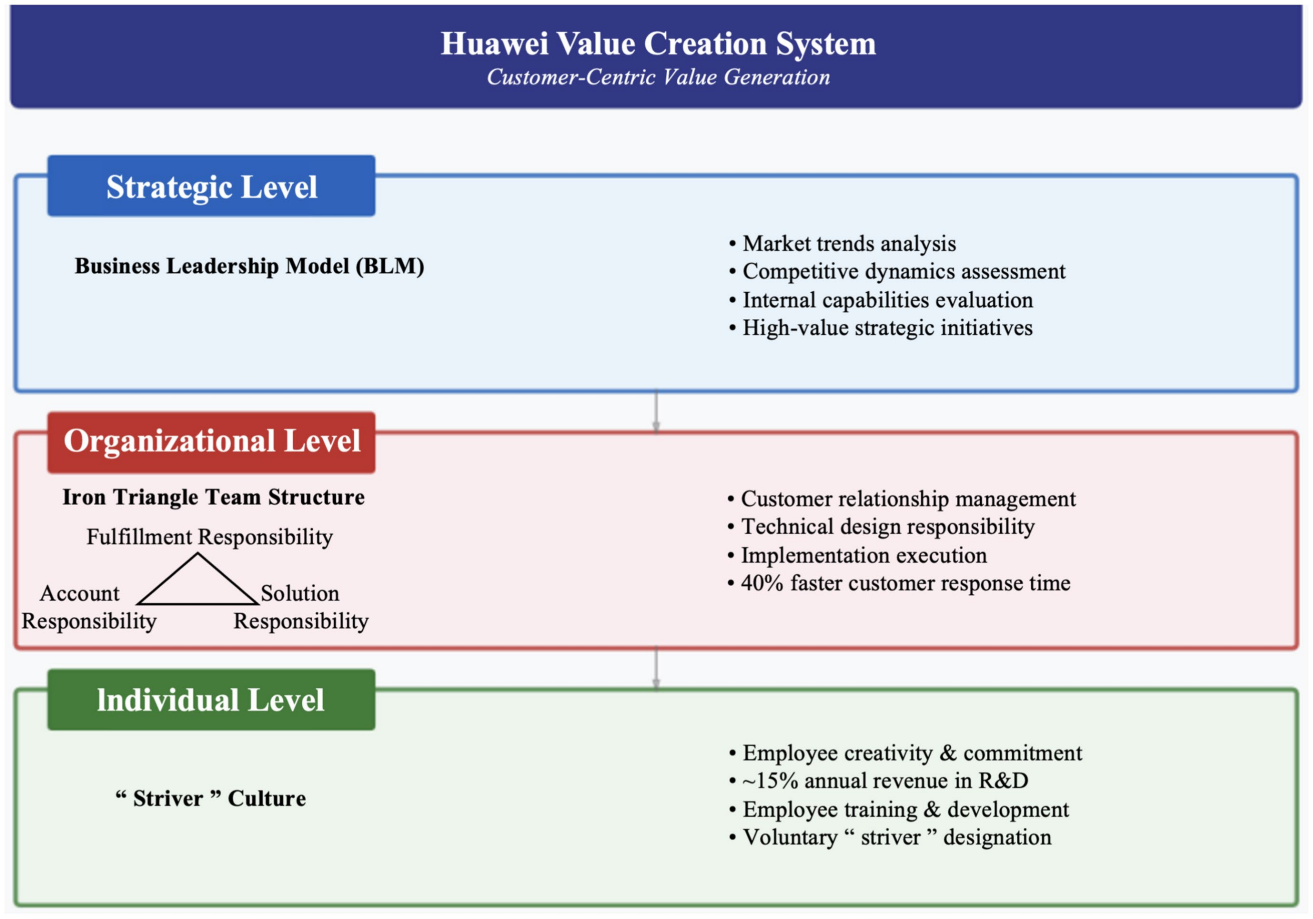
Huawei’s value creation system.

Huawei’s approach to value creation is fundamentally anchored in the principle of being “customer-centric.” This orientation, established in the Huawei Basic Law of 1998, directs all organizational activities toward creating value for customers, with the understanding that customer value creation ultimately generates returns for all stakeholders including employees (see Figure 2).

The company’s value creation system operates at three interconnected levels: strategic, organizational, and individual. At the strategic level, Huawei employs the Business Leadership Model (BLM) to align organizational capabilities with market opportunities. The BLM framework facilitates systematic analysis of market trends, competitive dynamics, and internal capabilities, enabling the company to identify high-value strategic initiatives and allocate resources accordingly. This strategic discipline ensures that value creation efforts remain focused on areas with the greatest potential for customer impact and competitive differentiation.

At the organizational level, Huawei has developed the “Iron Triangle” team structure to enhance customer-facing value creation. Each Iron Triangle comprises three roles: Account Responsibility, responsible for customer relationships; Solution Responsibility, responsible for technical design; and Fulfillment Responsibility, responsible for implementation execution. This integrated team structure ensures that customer needs are comprehensively addressed while facilitating rapid response to market requirements. Interview data indicates that the Iron Triangle structure has enabled Huawei to achieve 40% faster customer response times compared to competitors using traditional functional structures.

At the individual level, Huawei emphasizes “striver” culture as the foundation of value creation. The company explicitly recognizes that in knowledge-intensive industries, employee creativity, commitment, and effort constitute the primary source of value creation. To cultivate and sustain this value-creating capacity, Huawei invests approximately 15% of annual revenue in research and development, with specific investments growing from CNY 3.9 billion in 2004 to CNY 192.3 billion in 2025, representing a cumulative R&D expenditure exceeding CNY 1382 billion over the past decade (Huawei Annual Report, 2025). This sustained investment reflects the company’s long-term commitment to capability building rather than short-term profit maximization, with significant portions allocated to employee training and development. The company’s “striver” designation, which employees must voluntarily apply for and commit to, establishes clear expectations for work intensity and dedication in exchange for enhanced rewards and opportunities.

The integration of these three levels creates a coherent value creation system in which strategic priorities guide organizational arrangements, which in turn shape individual contributions. This alignment ensures that value creation efforts are both focused and mutually reinforcing, generating sustainable competitive advantage through continuous innovation and customer responsiveness.

Observational evidence from consulting engagements further illustrates how value creation is structured around collective effort rather than individual authority. In observed Iron Triangle team meetings, decisions regarding customer solutions were made collectively through structured deliberation processes involving all three roles (Account Responsibility, Solution Responsibility, Fulfillment Responsibility), with no single individual having unilateral decision authority. This collective decision-making structure ensures that customer solutions integrate diverse perspectives and that knowledge is shared across functional boundaries rather than hoarded by individual experts. For example, in one observed meeting regarding a complex telecommunications infrastructure project, the Solution Responsibility identified technical constraints, the Fulfillment Responsibility highlighted implementation challenges, and the Account Responsibility provided customer context, resulting in a jointly developed solution that none of the individual roles could have created independently. This collaborative approach to value creation builds organizational capability that persists even when individual team members transition to other roles or leave the company.

Analytical Insight: The sustainability of Huawei’s value creation system derives from several key mechanisms. First, the customer-centric orientation creates an external focus that prevents internal politics and resource misallocation, ensuring that employee efforts consistently generate market-validated value. Second, the Iron Triangle structure distributes authority and accountability across functional roles, reducing dependence on individual leaders and building collective organizational capability. Third, the striver culture operates as a self-selection mechanism that attracts employees committed to long-term value creation while naturally filtering out those seeking short-term transactional employment. These mechanisms collectively ensure that value creation is not dependent on any single individual or transaction but is embedded in organizational systems and culture, thereby contributing to sustainable competitive advantage through human capital development.

Interview evidence reveals the practical implementation of this philosophy: “Our value creation starts and ends with the customer. Every decision we make, every resource we allocate, must ultimately serve the customer. The striver culture is not just about working hard; it’s about working smart in directions that create genuine customer value” (Interview 1, Former Senior HR Executive, 2021). This customer-centric orientation is reinforced through concrete organizational practices and resource allocation decisions.

### 4.2 Value evaluation: result-oriented assessment system

**Figure 3:**
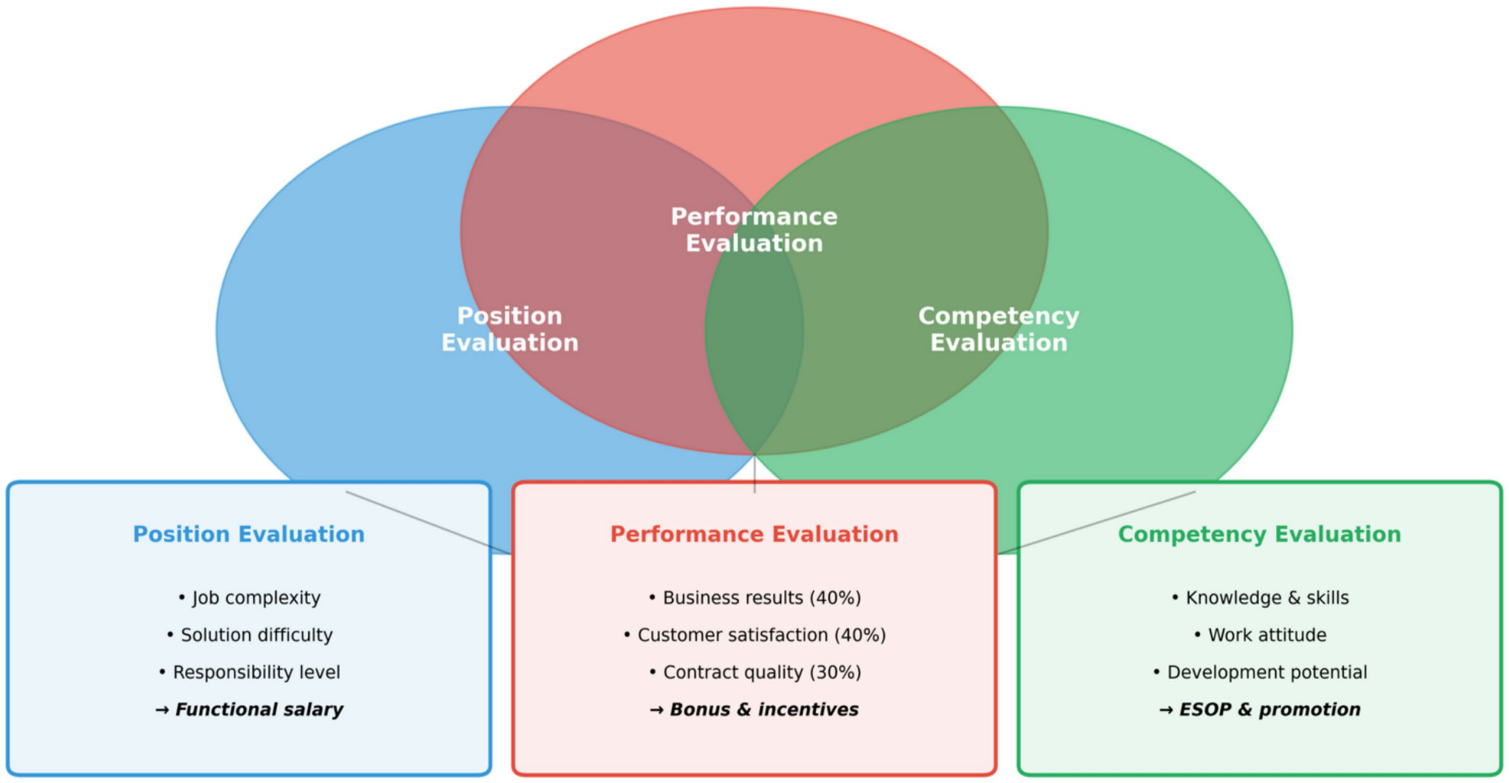
Huawei’s three-dimensional value evaluation system.

Huawei’s value evaluation system, characterized as “result-oriented,” provides comprehensive mechanisms for assessing value creation across multiple dimensions. The system, often referred to internally as the “Three Rulers” model, integrates position evaluation, performance assessment, and competency review to create a holistic picture of employee contributions (see Figure 3).

Position evaluation assesses the relative value of different organizational roles based on three criteria: job complexity, difficulty of solutions required, and level of responsibility. This evaluation establishes the baseline contribution expected from each position, enabling fair comparisons across different functions and levels. The position evaluation system uses a point-factor methodology adapted from Hay Group’s job evaluation framework, with modifications to reflect Huawei’s specific business context and cultural values. Positions are classified into grades that determine base salary ranges and eligibility for various incentive programs.

Performance evaluation at Huawei extends beyond traditional metrics to incorporate multiple dimensions of contribution. The company’s performance assessment framework weights business results at 40%, customer satisfaction at 40%, and contract quality (including gross margin and payment terms) at 30%. This multi-dimensional approach prevents the common problem of prioritizing revenue growth over profitability and customer relationships. Interview respondents emphasized that this balanced scorecard approach has been instrumental in maintaining sustainable growth rather than pursuing short-term volume at the expense of long-term viability. A distinctive feature of Huawei’s performance evaluation is the forced distribution system, which requires that performance ratings follow a predetermined curve: approximately 10% of employees receive top ratings (A), 25% receive above-average ratings (B+), 40% receive satisfactory ratings (B), 20% receive below-expectations ratings (C), and 5% receive unsatisfactory ratings (D). While controversial, this system ensures that performance differentiation is maintained even in growing organizations where absolute performance improvements might otherwise mask relative differences.

Competency evaluation assesses employee capabilities across dimensions including knowledge, skills, work attitude, and development potential. Huawei has developed detailed competency models for different job families, specifying the behavioral indicators associated with various proficiency levels. Competency assessments inform decisions about promotions, job assignments, and development needs, ensuring that employees are placed in positions where their capabilities can generate maximum value.

The integration of these three evaluation dimensions creates a comprehensive assessment system that captures different aspects of employee contribution. Position evaluation establishes baseline expectations, performance evaluation measures actual results, and competency evaluation assesses underlying capabilities. Together, they provide the information necessary for fair and effective value distribution decisions.

Concrete examples from corporate documents illustrate how evaluation results directly influence career decisions. In Huawei’s 2022 HR report, the company documented that 78% of promotion decisions were directly informed by competency assessment results, with specific competency profiles developed for over 200 job families. For instance, the technical competency model for 5G network engineers specifies five proficiency levels across 12 competency dimensions, including technical knowledge depth, problem-solving complexity, innovation contribution, and knowledge transfer effectiveness. Employees must demonstrate behavioral indicators at specified levels to qualify for promotion, ensuring that advancement is based on demonstrated capability rather than tenure or relationships. This systematic approach to competency-based career development ensures that the organization continuously builds the human capital capabilities required for long-term success while providing employees with clear development pathways.

Observational evidence from consulting engagements further illustrates how the Three Rulers evaluation system operates in practice to maintain objectivity and prevent rating inflation. In observed performance calibration sessions, divisional managers systematically reviewed employee cases across the three evaluation dimensions rather than relying on single metrics or general impressions. During one observed calibration meeting for a product development division, managers spent over 3 hours discussing borderline cases, particularly debating whether an engineer with exceptional technical achievement but declining teamwork indicators deserved a B + or B rating. The group ultimately maintained the B rating based on explicit competency model criteria, demonstrating that the forced distribution system is not merely a numerical exercise but involves substantive deliberation about multidimensional contribution. This rigorous calibration process ensures that performance differentiation reflects genuine variation in contribution rather than manager leniency or team politics. For example, in one observed annual competency review panel for senior technical staff, assessors required candidates to present documented evidence of knowledge transfer, including specific instances where they had mentored junior engineers and shared proprietary methodologies. One candidate with strong technical performance was deferred for promotion when assessors found that while they had solved complex problems independently, they had failed to document or transfer that knowledge to team members, thereby violating the competency model’s explicit behavioral indicator for “building organizational capability.” This case illustrates how competency evaluation translates abstract criteria into concrete assessment decisions that shape career trajectories and reinforce the expectation that sustainable contribution requires developing collective capability rather than merely individual expertise.

Analytical Insight: The Three Rulers evaluation system contributes to sustainable HRM through several interconnected mechanisms. The multi-dimensional assessment (business results, customer satisfaction, contract quality) prevents short-term gaming behaviors by requiring balanced performance across multiple stakeholders. The forced distribution system maintains performance differentiation even during periods of organizational growth, preventing the “grade inflation” that often undermines evaluation effectiveness in expanding companies. Perhaps most importantly, the separation of position evaluation, performance evaluation, and competency evaluation creates a nuanced understanding of employee contribution that supports both immediate reward decisions and long-term development planning. This comprehensive approach ensures that evaluation serves not merely as a control mechanism but as a developmental tool that identifies strengths to build and gaps to address, thereby supporting sustainable human capital development.

A senior HR director elaborated on the rationale behind this multi-dimensional approach: “Performance evaluation at Huawei is not only results-oriented but also closely linked to long-term value creation. We evaluate not just what you achieved, but how you achieved it and what capabilities you built. The 40–40-30 weighting ensures that no single metric dominates, preventing short-term gaming behaviors that might undermine sustainable development” (Interview 3, Former HR Director, 2022). This evaluation philosophy directly supports sustainable HRM by aligning performance measurement with long-term value creation rather than short-term transactional outcomes.

### 4.3 Value distribution: striver-focused reward system

**Figure 4:**
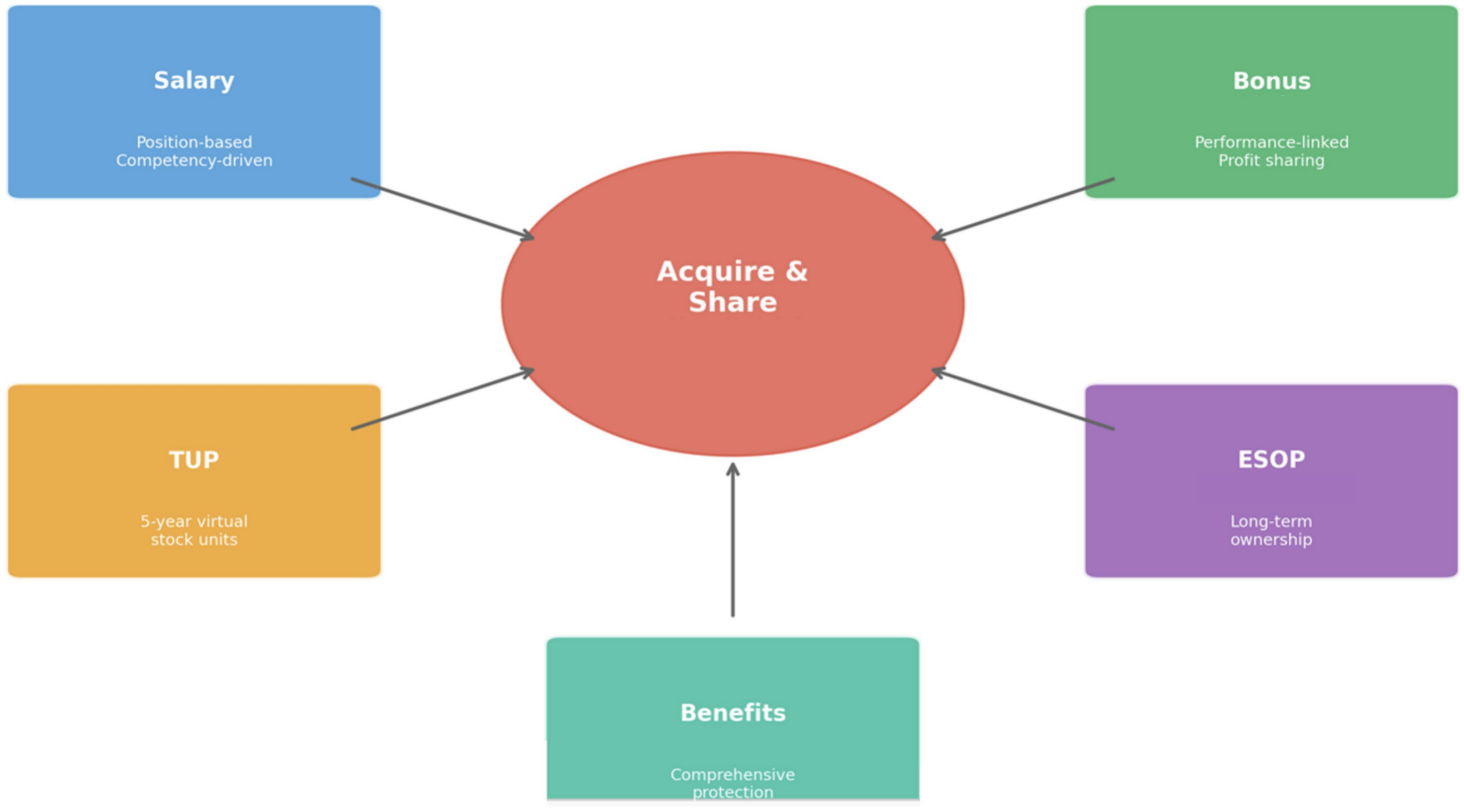
Huawei’s value distribution: the “Acquire and Share” system.

Huawei’s value distribution system embodies the principle of being “striver-focused,” ensuring that employees who contribute most to organizational success receive proportionately greater rewards. The company’s “Acquire and Share” distribution philosophy establishes that rewards should be earned through contribution rather than granted based on seniority or position alone (see Figure 4).

The distribution system comprises multiple components designed to address different employee needs and time horizons. Base salary, determined by position grade and individual competency, provides income stability and reflects the market value of employee capabilities. Huawei maintains competitive base salaries, typically positioning at the 75th percentile of relevant labor markets to attract and retain high-quality talent.

Performance bonuses constitute a significant portion of total compensation, with individual awards linked to organizational, team, and personal performance. At the organizational level, Huawei distributes a portion of company profits to business units based on their contributions, with the total bonus pool fluctuating with company performance. This mechanism ensures that employees share in organizational success while also bearing collective responsibility for results. In 2023, Huawei distributed approximately 65% of global profits to employees through various incentive programs. This substantial profit-sharing ratio, consistently maintained above 60% over the past decade (Huawei Annual Reports, 2014–2023), demonstrates the company’s commitment to its employee-ownership model and provides concrete evidence of the “Acquire and Share” philosophy in practice. The total employee compensation package, including base salary, bonuses, and stock-based incentives, positions Huawei at the 75th percentile or above in relevant labor markets, supporting talent attraction and retention objectives.

The Time-based Unit Plan (TUP) represents an innovative medium-term incentive mechanism. TUP grants employees virtual stock units that appreciate with company value over a five-year period, providing annual dividend equivalents and terminal value appreciation. Unlike traditional stock options, TUP units expire after five years, preventing the accumulation of passive wealth and ensuring that incentives remain tied to ongoing contribution. Currently, TUP covers more than 80,000 core employees, creating a broad-based ownership culture without diluting actual equity.

The Employee Stock Ownership Plan (ESOP) provides long-term incentives for senior contributors and high-potential employees. Huawei is 100% employee-owned, with founder Ren Zhengfei holding less than 1% of shares while the remaining equity is distributed among approximately 120,000 employees. This ownership structure aligns employee interests with long-term organizational success while reinforcing the principle that value creators should be value owners.

Beyond financial rewards, Huawei’s distribution system includes comprehensive benefits, recognition programs, and development opportunities. Benefits include healthcare, housing assistance, and family support programs designed to address employee needs and reduce life concerns that might distract from work performance. Recognition programs celebrate exceptional contributions through public acknowledgment, awards, and opportunities to participate in high-visibility projects. Development opportunities, including training, international assignments, and challenging job rotations, are allocated preferentially to high performers, creating a virtuous cycle in which contribution leads to capability development that enables further contribution.

Corporate documents provide specific examples of TUP’s motivational effects. According to Huawei’s (2023) HR report, TUP coverage expanded from 60,000 employees in 2018 to over 80,000 core employees in 2023, with the average TUP grant value increasing by 35% over this period. The five-year vesting structure creates a “golden handcuff” effect that retains key talent during critical project phases. For example, senior engineers working on 5G infrastructure development typically receive TUP grants that vest over the 3–5 year development cycle, ensuring their continued engagement through product completion. The 2023 report noted that among employees with 5 + years of TUP participation, voluntary turnover was 62% lower than among employees with less than 2 years of participation, demonstrating the retention effectiveness of medium-term incentives. This temporal alignment between incentive structures and business development cycles ensures that human capital investments generate returns before key talent departs.

Observational evidence from consulting engagements further illustrates how the striver-focused distribution system differentiates rewards based on contribution in actual allocation processes. In observed compensation committee meetings, bonus pool distributions were explicitly tied to the forced distribution ratings generated through the Three Rulers system, with committee members reviewing unit-level performance data and individual evaluation outcomes before approving allocations. During one observed meeting for a wireless network business unit, the committee allocated performance bonuses ranging from 1.8 months to 11 months of base salary, with the top-rated performers receiving awards six times larger than satisfactory-rated employees in the same role grade. This visible differentiation in reward magnitude, communicated through transparent calculation formulas shared with employees, reinforced the principle that distribution follows evaluation outcomes rather than tenure or position alone. This transparent linkage between evaluation and distribution ensures that employees clearly perceive the connection between their assessed contributions and their received rewards. For example, in one observed TUP eligibility review meeting, committee members explicitly screened candidates against both performance ratings and striver designation status, rejecting several long-tenured employees with satisfactory performance but without active striver commitment. Conversely, a relatively junior engineer with three consecutive A ratings and demonstrated willingness to accept challenging assignments in Africa and Southeast Asia was granted TUP units exceeding those of some senior colleagues. This observed allocation pattern demonstrates that the “Acquire and Share” philosophy prioritizes sustained high contribution and organizational commitment over seniority, ensuring that value distribution consistently flows to those who create disproportionate value regardless of organizational rank or career stage.

Analytical Insight: The sustainability of Huawei’s distribution system lies in its multi-layered approach to addressing employee needs across different time horizons and career stages. The base salary provides immediate income security, performance bonuses reward recent contributions, TUP creates medium-term incentives that retain core talent over 5-year cycles, and ESOP aligns employee interests with long-term organizational survival beyond any individual’s career horizon. This temporal diversification of rewards ensures that employees at different career stages find meaningful incentives to maintain high performance and commitment. Furthermore, the substantial magnitude of potential rewards through TUP and ESOP creates opportunity costs for leaving that exceed what competitors typically offer, thereby reducing turnover among high performers who have accumulated substantial virtual equity. The 100% employee-owned structure, with founder Ren Zhengfei holding less than 1% of shares, ensures that value creators are also value owners, creating a genuine partnership relationship that transcends traditional employment contracts and supports sustainable organizational development.

The compensation director explained the underlying philosophy: “We believe that those who create value should share in the value. Our distribution system is designed to reward strivers, not seniority or position alone. The ‘Acquire and Share’ principle ensures that rewards are earned through contribution, creating a direct link between individual effort and organizational success” (Interview 5, Compensation Director, 2021). This principle is operationalized through multiple mechanisms that address different time horizons and employee needs.

### 4.4 The integrated value management cycle

The sustainable HRM impact of Huawei’s value management system derives not from any single component but from their integration into a coherent, self-reinforcing cycle. The relationships between value creation, evaluation, and distribution create feedback loops that continuously align employee behavior with organizational objectives while fostering long-term capability development.

Value creation provides the foundation for the entire system by establishing what constitutes valuable contribution. Huawei’s customer-centric orientation clearly signals that value is measured by customer impact rather than internal activities or personal effort. This clarity enables employees to focus their efforts on activities that genuinely create value, reducing wasted effort on peripheral activities. The Iron Triangle structure and striver culture further reinforce this focus by creating organizational arrangements and cultural expectations that support customer-focused value creation.

Value evaluation translates value creation into measurable assessments that enable fair distribution. The Three Rulers evaluation system captures multiple dimensions of contribution, ensuring that distribution decisions reflect a comprehensive understanding of employee value. By separating position evaluation (establishing baseline expectations), performance evaluation (measuring actual results), and competency evaluation (assessing underlying capabilities), the system provides nuanced information that supports differentiated distribution decisions.

Value distribution completes the cycle by rewarding contribution in ways that motivate future value creation. The Acquire and Share philosophy ensures that rewards are tied to contribution, creating powerful incentives for high performance. The multi-component distribution system addresses different employee needs and time horizons, ensuring that rewards remain meaningful across diverse employee populations and career stages. Perhaps most importantly, the substantial magnitude of potential rewards—particularly through TUP and ESOP—creates powerful motivation for sustained high performance.

The cycle’s sustainability derives from its ability to balance short-term performance with long-term capability development. While performance-based rewards motivate immediate results, competency evaluation and development opportunities ensure that employees continue building capabilities that will generate future value. The ownership structure created through ESOP and TUP aligns employee time horizons with organizational longevity, encouraging decisions that balance immediate returns with sustainable growth.

Table 1 summarizes the key differences between traditional HRM approaches and the value management cycle approach to sustainable HRM. And, Table 2 summarizes key components of Huawei’s value management system.

**Table 1:** Comparison of traditional HRM and value management cycle HRM.

Dimension	Traditional HRM	Value management cycle HRM
Core philosophy	Cost control and efficiency	Value creation and sharing
Value creation focus	Position-based duties	Customer-centered outcomes
Evaluation criteria	Seniority and qualifications	Results and contributions
Distribution principle	Equal distribution emphasis	Differential reward system
Employee relationship	Employment contract	Partnership and co-ownership
Sustainability orientation	Short-term performance	Long-term sustainable development

**Table 2:** Key components of Huawei’s value management system.

Component	Mechanism	Key features	Sustainability outcome
Value creation	Strategic decoding	Align individual goals with company strategy	Strategic alignment and focus
	Iron triangle teams	Cross-functional customer-facing teams	Customer value maximization
	R&D investment	15% of revenue allocated to innovation	Continuous innovation capability
Value evaluation	Position evaluation	Job complexity and responsibility assessment	Fair internal value positioning
	Performance assessment	Business results + customer satisfaction	Results-driven culture
	Competency review	Knowledge, skills, and attitude evaluation	Employee development and growth
Value distribution	Functional salary	Position and competency-based pay	Internal equity and motivation
	Performance bonus	Profit sharing linked to performance	Immediate reward for contributions
	Employee stock ownership	Long-term incentive for core contributors	Long-term commitment and retention

## 5 Discussion

### 5.1 Theoretical implications

This study contributes to sustainable HRM theory in several important ways. First, it provides an integrative framework that synthesizes fragmented sustainable HRM research into a coherent system. While previous studies have examined individual sustainable HRM practices such as green HRM, ethical leadership, or employee well-being initiatives, this research demonstrates how these practices can be systematically integrated through the value management cycle (Aust et al., 2020; Katper et al., 2026). The framework positions sustainable HRM not as a collection of separate initiatives but as an interconnected system in which value creation, evaluation, and distribution mutually reinforce each other.

Second, the study extends the Resource-Based View of HRM by demonstrating how human capital advantages can be sustained through systematic value management. While RBV theory identifies human capital as a potential source of competitive advantage, it provides limited guidance on how organizations can develop and maintain such advantages over time (Barney, 1991). The value management cycle framework addresses this gap by specifying the mechanisms through which organizations can continuously cultivate, evaluate, and reward human capital contributions in ways that sustain competitive advantage.

Third, the research contributes to understanding how sustainable HRM can be implemented in emerging economy contexts. Much of the existing sustainable HRM literature draws from Western developed economy contexts, potentially limiting its applicability to different institutional and cultural environments (Faisal and Naushad, 2020). Huawei’s experience demonstrates that sustainable HRM principles can be effectively adapted to Chinese institutional conditions while maintaining theoretical coherence. The company’s success suggests that the value management cycle framework may have broader applicability across different national and cultural contexts.

Fourth, the study advances understanding of the relationship between HRM and organizational sustainability. Previous research has often focused on the environmental dimensions of sustainability, with less attention to how HRM practices can contribute to long-term organizational viability (Siddique et al., 2026). The value management cycle framework explicitly addresses this relationship by demonstrating how HRM practices can be designed to balance short-term performance with long-term capability development and employee well-being.

### 5.2 Practical implications

The findings of this study offer several practical implications for organizational leaders and HR professionals seeking to implement sustainable HRM systems. First, the research underscores the importance of systematic integration in HRM design. Rather than implementing isolated best practices, organizations should develop coherent HRM systems in which value creation, evaluation, and distribution mechanisms are mutually aligned and reinforcing. This integration requires careful attention to the signals sent by different HRM practices and their combined effects on employee motivation and behavior.

Second, the study highlights the critical role of value clarity in sustainable HRM. Huawei’s experience demonstrates that clear articulation of what constitutes value—specifically, customer-centered outcomes—provides the foundation for effective evaluation and distribution systems. Organizations should invest time in defining and communicating their value creation logic, ensuring that employees understand how their contributions generate organizational value.

Third, the research suggests that sustainable HRM requires substantial investment in employee development. Huawei’s allocation of 15% of revenue to R&D and significant additional resources to training reflects a recognition that human capital advantages must be continuously renewed. Organizations seeking to implement sustainable HRM should be prepared to make similar investments, viewing employee development not as a cost to be minimized but as an investment in future value creation capacity.

Fourth, the study demonstrates the importance of differential reward systems in motivating high performance. Huawei’s approach of “giving the locomotive a full tank of oil” ensures that top contributors receive rewards proportionate to their contributions. While such differentiation may create temporary tensions, the long-term effect is to attract and retain high performers while motivating continuous improvement among all employees.

Fifth, the research highlights the value of employee ownership in aligning interests and time horizons. Huawei’s ESOP and TUP programs create a genuine ownership culture in which employees benefit directly from organizational success. Organizations seeking to implement sustainable HRM should consider how various forms of shared ownership might be adapted to their specific contexts and regulatory environments.

### 5.3 Limitations and future research

This study has several limitations that should be acknowledged. First, as a single-case study focused on one company, the findings may have limited generalizability to other organizational contexts. While Huawei’s experience provides valuable insights, organizations in different industries, sizes, or institutional environments may face distinct challenges in implementing value management cycle HRM. Future research should examine how the framework can be adapted to different contexts, including small and medium enterprises, public sector organizations, and non-profit entities.

Second, the study relies primarily on qualitative data, which, while rich in contextual detail, limits the ability to make precise quantitative assessments of the relationships between HRM practices and outcomes. Future research could employ quantitative methods, such as surveys and longitudinal performance data, to more rigorously test the propositions derived from this study.

Third, the research focuses on the design and implementation of the value management cycle without fully addressing potential negative consequences or challenges. For example, Huawei’s forced distribution performance evaluation system has been criticized for potentially creating excessive internal competition and stress. Future research should examine the potential downsides of value management cycle practices and identify strategies for mitigating negative effects.

Fourth, the study does not fully address how the value management cycle might need to evolve in response to changing workforce demographics and expectations. As younger generations enter the workforce with different values and priorities, sustainable HRM systems may need to adapt to remain effective. Future research should examine how generational differences affect the design and implementation of value management cycle HRM.

Several directions for future research emerge from this study. First, comparative studies examining how different organizations implement value management cycle principles would enhance understanding of contextual factors affecting implementation success. Second, longitudinal research tracking the evolution of value management systems over time would illuminate how these systems adapt to changing organizational and environmental conditions. Third, research examining the relationship between value management cycle HRM and specific sustainability outcomes, such as employee well-being, innovation performance, and long-term financial results, would strengthen the empirical foundation for the framework.

## 6 Conclusion

This study has developed and illustrated the value management cycle as a comprehensive framework for sustainable HRM. Drawing on extensive analysis of Huawei’s pioneering HRM practices, the research demonstrates how value creation, evaluation, and distribution can be integrated into a coherent system that generates sustainable competitive advantage through human capital development.

The key findings of this study support several important conclusions. First, sustainable HRM requires systematic integration of HRM practices rather than implementation of isolated best practices. The value management cycle framework provides a theoretically grounded and practically proven approach to achieving such integration. Second, clear articulation of value creation logic—specifically, customer-centered value generation—provides the foundation for effective evaluation and distribution systems. Third, comprehensive evaluation systems that capture multiple dimensions of contribution enable fair and effective distribution decisions. Fourth, differential distribution systems that reward contribution proportionately create powerful motivation for sustained high performance. Huawei’s experience demonstrates that the value management cycle can be implemented at scale, supporting the management of nearly 200,000 employees across diverse global markets while maintaining high levels of organizational vitality and innovation capacity. The company’s success suggests that sustainable HRM is not merely an aspirational ideal but an achievable reality for organizations willing to make the necessary investments in system design and implementation.

The theoretical framework developed in this study contributes to sustainable HRM research by providing an integrative perspective that synthesizes fragmented literature into a coherent system. The framework extends the Resource-Based View of HRM by specifying mechanisms through which human capital advantages can be continuously cultivated and sustained. The research also contributes practical insights for organizational leaders and HR professionals seeking to implement sustainable HRM systems. As organizations navigate the challenges of the knowledge economy, the value management cycle offers a proven approach to aligning employee and organizational interests in pursuit of sustainable competitive advantage. By focusing on value creation, implementing objective evaluation systems, and distributing rewards equitably, organizations can develop the human capital necessary for long-term success while fostering employee well-being and commitment. The Huawei case demonstrates that this approach is not merely theoretical but has been successfully implemented at scale, offering valuable lessons for organizations worldwide.

## Data Availability

The original contributions presented in the study are included in the article/supplementary material, further inquiries can be directed to the corresponding author.
